# Step Process for Selecting and Testing Surrogates and Indicators of Afrotemperate Forest Invertebrate Diversity

**DOI:** 10.1371/journal.pone.0009100

**Published:** 2010-02-09

**Authors:** Charmaine Uys, Michelle Hamer, Rob Slotow

**Affiliations:** School of Biological and Conservation Sciences, University of KwaZulu-Natal, Pietermaritzburg, KwaZuluNatal, South Africa; Stanford University, United States of America

## Abstract

**Background:**

The diversity and complexity of invertebrate communities usually result in their exclusion from conservation activities. Here we provide a step process for assessing predominantly ground-dwelling Afrotemperate forest invertebrates' (earthworms, centipedes, millipedes, ants, molluscs) potential as surrogates for conservation and indicators for monitoring. We also evaluated sampling methods (soil and litter samples, pitfall traps, active searching quadrats and tree beating) and temporal (seasonal) effects.

**Methodology/Principal Findings:**

Lack of congruence of species richness across taxa indicated poor surrogacy potential for any of the focus taxa. Based on abundance and richness, seasonal stability, and ease of sampling, molluscs were the most appropriate taxon for use in monitoring of disturbance impacts. Mollusc richness was highest in March (Antipodal late summer wet season). The most effective and efficient methods were active searching quadrats and searching litter samples. We tested the effectiveness of molluscs as indicators for monitoring by contrasting species richness and community structure in burned relative to unburned forests. Both species richness and community structure changed significantly with burning. Some mollusc species (*e.g. Macroptychia africana*) showed marked negative responses to burning, and these species have potential for use as indicators.

**Conclusions/Significance:**

Despite habitat type (*i.e.*, Afrotemperate forest) being constant, species richness and community structure varied across forest patches. Therefore, in conservation planning, setting targets for coarse filter features (*e.g.*, habitat type) requires fine filter features (*e.g.*, localities for individual species). This is especially true for limited mobility taxa such as those studied here. Molluscs have high potential for indicators for monitoring, and this requires broader study.

## Introduction

A systematic approach to conservation includes both the measurement of biodiversity features for prioritizing areas, and adaptive management of these, including monitoring the impacts of management or disturbance [1]. Because of limited resources and potential impact on conservation assets, both inventories and monitoring need to be as effective and efficient as possible. It is impossible to sample and identify every species, even in small areas, and this is especially true for the hyperdiverse invertebrates [2]. Invertebrates may be important in terms of their relatively high levels of endemism [3], [4], and their responsiveness to environmental change [5] makes them potential indicators for monitoring (*e.g.* [6], [7]), and for conservation planning (for overview see [8]). Invertebrates are especially poorly represented in conservation activities, largely because of their enormous abundance and diversity, and the lack of appropriate information for many taxa [9], [10].

Protocols for monitoring biodiversity are not well established for terrestrial ecosystems [11], and more research is required on indicators [12]. Indicator taxa selected for monitoring must reflect environmental change and the reaction of other taxa [11], [13]. Most importantly, because of differential sensitivity of taxa to environmental disturbance, empirical studies are necessary to verify the appropriateness of a particular taxon as an indicator of disturbance [14]. A stepwise, integrated, approach is necessary to properly identify biodiversity surrogates or indicators [6], [15]. The following steps are recommended: (1) a survey using standardised, quantified effort [2], [16]; (2) an assessment of potential taxa for use in conservation planning or monitoring [6 and references therein], [15]; and (3) a test of the selected indicator taxon for monitoring a particular disturbance [6], [15]). Rohr *et al.* [15] add in the additional dimension of testing which sampling method is most appropriate for a particular chosen taxon.

The target taxa for this study, predominantly ground-dwelling, flightless invertebrates, are potentially important for biodiversity assessment and monitoring. Invertebrates such as molluscs, earthworms, centipedes, millipedes, and onychophorans may be suitable surrogate taxa for biodiversity assessments because they have limited dispersal ability and consequently they may exhibit high levels of endemism [4], [17]–[22], and ants have been widely recommended as surrogates and indicators [23]–[26]. These taxa have relatively well known taxonomy, and are easily observed, and they may also be suitable for monitoring disturbance because they do not have complex life cycles (except for ants), adults are relatively long-lived compared to most insects [7], and because they have limited mobility they are less likely to escape and colonise other habitats after disturbance.

The aim of this study was to assess the potential for these taxa to serve as surrogates for biodiversity assessment, and as indicators for monitoring disturbance. We undertook this study in Afrotemperate forest [27] in the KwaZulu-Natal Drakensberg, South Africa, where forest patches have been small and fragmented since the last glacial maximum (18 000 y.b.p.), and have expanded and contracted prior to that [see 28 for forest history]. The objectives were: (1) to compare species richness and community structure across seasons to identify the most suitable time of year for diversity assessment and monitoring of the target taxa, and to identify taxa that do not show marked seasonal changes in diversity; (2) to determine which sampling methods used to determine species richness were most effective and efficient for use in biodiversity assessment and monitoring for the target taxa, in different months; (3) to determine which flightless invertebrate taxa were most suitable for use in biodiversity assessment (surrogates) and monitoring (indicators) using the approach and criteria of Summerville *et al.* [6], but including the additional component of endemism; and (4) to experimentally test these recommendations for use in monitoring, using burned and unburned Afrotemperate forest patches at a different study site. Note that fire in Afrotemperate forests is becoming an increasing conservation concern (*e.g.* [29]).The study also illustrates the process that should be undertaken to evaluate the taxa selected for a large scale survey or a monitoring programme, before such activities are implemented on a large scale.

## Results

We collected 4275 individual specimens representing 55 species in the four months from the three sites at Injisuthi (Table 1). The 55 species comprised 26 mollusc, four earthworm, one onychophoran, six centipede, 11 millipede and seven ant species. Because only a single onychophoran species was collected, this group was excluded from further analyses.

**Table 1:** Mollusc, earthworm, onychophoran, centipede, millipede and ant species collected during seasonal sampling at Injisuthi (abundance data).

Order	Family	Species	M	J	S	D
**Class Gastropoda**						
Neritopsina	Hydrocenidae	*Hydrocena noticola* Benson, 1856	169	168	334	169
Architaenioglossa	Cyclophoridae	*Chondrocyclus isipingoensis (Sturany, 1898)	25	54	29	34
Eupulmonata	Pupillidae	*Lauria dadion (Benson, 1864)	12	9	5	7
Eupulmonata	Orculidae	*Fauxulus glanvilleanus (darglensis) (Ancey, 1888)	23	36	88	50
Eupulmonata	Orculidae	*Fauxulus mcbeanianus Melville and Ponsonby, 1901	15	17	25	56
Eupulmonata	Orculidae	***Fauxulus* sp.**	**5**	**0**	**0**	**0**
Eupulmonata	Vertiginidae	*Pupisoma harpula* (Reinhardt, 1886)	5	0	0	14
Eupulmonata	Vertiginidae	*Truncatellina sykesii* (Melville and Ponsonby, 1893)	60	50	67	35
Eupulmonata	Clausiliidae	*Macroptychia africana (Melville and Ponsonby, 1899)	15	12	13	13
Eupulmonata	Achatinidae	***Archacatina* sp.**	**0**	**0**	**0**	**13**
Eupulmonata	Streptaxidae	*Gulella mariae (Melville and Ponsonby, 1892)	10	12	8	9
Eupulmonata	Valloniidae	*Acanthinula* sp.	35	0	1	24
Eupulmonata	Charopidae	*Afrodonta novemlamellaris* (Burnup, 1912)	10	11	0	6
Eupulmonata	Charopidae	*Trachycystis contabulata Connolly, 1932	11	65	46	33
Eupulmonata	Charopidae	*Trachycystis ectima (Melville and Ponsonby, 1899)	0	13	14	25
Eupulmonata	Charopidae	*Trachycystis glanvilliana (Ancey, 1893)	0	2	3	1
Eupulmonata	Charopidae	*Trachycystis rudicostata* Connolly, 1923	94	91	51	31
Eupulmonata	Charopidae	*Trachycystis subpinguis Connolly, 1922	**0**	**0**	**0**	**14**
Eupulmonata	Charopidae	*Trachycystis venatorum Connolly, 1932	**23**	**0**	**0**	**0**
Eupulmonata	Helicarionidae	*Kaliella euconuloides Melville and Ponsonby, 1908	26	8	29	117
Eupulmonata	Euconulidae	*Afroconulus diaphanus* (Connolly, 1922)	10	5	7	4
Eupulmonata	Achatinidae	*Archacatina dimidiata (Smith, 1878)	0	2	1	1
Eupulmonata	Vertiginidae	*Pupisoma orcula* (Benson, 1850)	14	63	86	0
Eupulmonata	Pupillidae	***?Pupilla fontana* (Krauss, 1848)**	**3**	**0**	**0**	**0**
Eupulmonata	Charopidae	***Trachycystis* sp.**	**1**	**0**	**0**	**0**
Eupulmonata	Chlamydephoridae	*Chlamydephorus burnupi Smith, 1892	**2**	**0**	**0**	**0**
**Class Oligochaeta**						
Haplotaxida	Acanthodrilidae	*Dichogaster* sp.	0	0	0	4
Haplotaxida	Acanthodrilidae	*Parachilota* sp. 1	0	0	0	29
Haplotaxida	Acanthodrilidae	*Parachilota* sp. 2	3	0	0	1
Opisthopora	Microchaetidae	*Proandricus* sp.	0	0	0	3
**Class Onychophora**						
Onychophora	Onychophora	*Opisthopatus cinctipes Purcell, 1899	1	0	0	0
**Class Chilopoda**						
Geophilomorpha	Geophilidae	*Rhysida afra (afra)	0	0	0	11
Geophilomorpha	Geophilidae	sp. 2	0	0	0	36
Geophilomorpha	Geophilidae	sp. 1	8	7	1	20
Lithobiomorpha	Henicopidae	*Paralamyctes spenceri	4	5	3	1
Lithobiomorpha	Henicopidae	*Lamyctes africana*	2	0	0	92
Lithobiomorpha	Henicopidae	*Lamyctes* sp.	3	0	0	1
**Class Diplopoda**						
Sphaerotheriida	Sphaerotheriidae	*Sphaerotherium dorsale* (Gervais, 1847)	2	3	63	23
Sphaerotheriida	Sphaerotheriidae	**Sphaerotherium mahaium Schubart, 1958	75	27	26	180
Sphaerotheriida	Sphaerotheriidae	*Sphaerotherium* sp.	0	0	12	19
Polydesmida	Dalodesmidae	*Gnomeskelus attemsii* Verhoeff, 1939	1	1	0	23
Polydesmida	Dalodesmidae	*Gnomeskelus montivagus* Verhoeff, 1939	1	6	2	27
Polydesmida	Dalodesmidae	*Gnomeskelus* sp.	27	5	7	74
Polydesmida	Gomphodesmidae	*Ulodesmus simplex Lawrence, 1953	4	7	18	32
Spirostreptida	Odontopygidae	*Spinotarsus* sp. 2	1	0	16	1
Spirostreptida	Odontopygidae	*Spinotarsus* sp. 1	1	1	0	3
Spirostreptida	Spirostreptidae	**Doratogonus montanus Hamer, 2000	2	2	0	3
**Class Insecta**						
Hymenoptera	Formicidae	sp. 1	3	0	1	0
Hymenoptera	Formicidae	sp. 2	9	0	2	520
Hymenoptera	Formicidae	sp. 3	20	0	18	18
Hymenoptera	Formicidae	sp. 4	5	0	0	6
Hymenoptera	Formicidae	sp. 5	7	12	17	16
Hymenoptera	Formicidae	sp. 6	8	1	14	0
Hymenoptera	Formicidae	sp. 7	16	1	4	0

M  =  March (autumn), J  =  June (winter), S  =  September (spring), and D  =  December (summer). Bold species are those that were sampled in a single season only. *  =  endemic to South Africa; **  =  endemic to the Drakensberg region.

### Seasonal Changes in Richness and Community Structure

**Figure 1:**
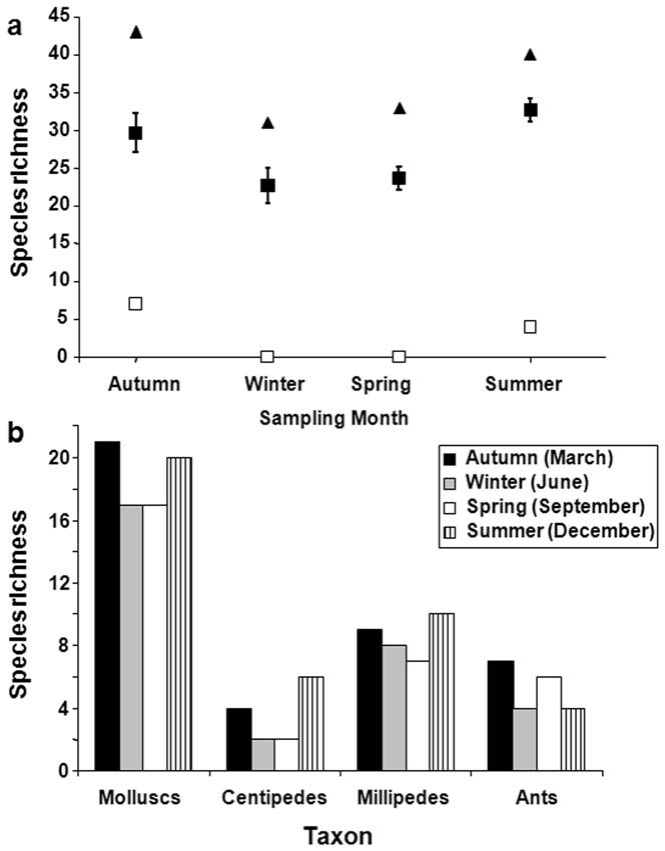
The influence of season on species richness to determine suitability for use in biodiversity assessment and monitoring. (A) All taxa combined: total species richness (triangles), mean species richness ± one standard deviation (solid squares) and unique species (open squares); and (B) taxa separately. Data are for three forests combined.

Of the 55 species, 22 (40%) were recorded in all four seasons, 11 in three of the four seasons, seven in two of the four seasons and 11 species (20%) were collected in one season only (Table 1). Nineteen species (35%) were collected only in the two wetter (and warm) months (December and March) and no species were unique to the dry season (June and September) (Table 1), which suggests that the flightless invertebrate community in winter is merely a subset of the summer wet season community. Total species richness, mean species richness, and unique species richness for all taxa was lower in the cool, dry season (June and September) compared to the warm, wet season (March and December) (Fig. 1A). When assessing taxa separately, species richness of molluscs, centipedes, and millipedes was slightly higher in wet season months (March and December) than dry season months, but only by a couple of species (Fig. 1B). Mollusc species richness ranged across the four seasons from 14 to 18, 12 to 15, and 15 to 17 species respectively for the three different forests. Millipede richness ranged from 2 to 8, 6 to 7, and 5 to 9 respectively for the three forests across the seasons.

**Figure 2:**
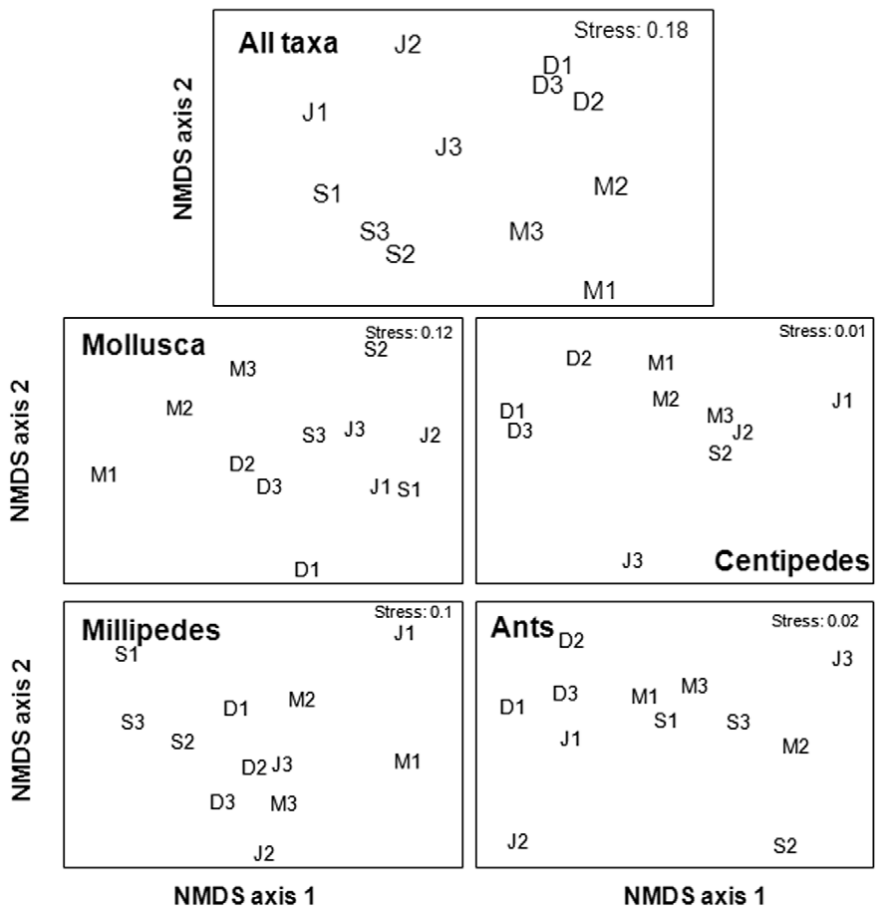
Influence of season on community structure of invertebrates. (A) all taxa; (B) molluscs; (C) centipedes; (D) millipedes; and (E) ants illustrated using multidimensional scaling (MDS). Letters indicate sampling season (M  =  March (autumn), J  =  June (winter), S  =  September (spring) and D  =  December (summer)), and numbers the site (forest).

Community structure was significantly different between March and June (ANOSIM: R  =  0.852), March and September (R  =  0.815), March and December (R  =  0.778), June and December (R  =  0.778), and September and December (R  =  0.963) (Fig. 2A). However, the community did not differ between the two dry season months (June and September) (R  =  0.278). There was a significant difference in mollusc community structure between autumn (March) and winter (June) (R  =  0.889), autumn (March) and spring (September) (R  =  0.796), and winter (June) and summer (December) (R  =  0.944), but the community differed less between autumn (March) and summer (December) (R  =  0.519) (Fig. 2B). Mollusc community structure was similar between the two dry season months (June and September) (R = −0.074). Centipedes showed distinct temporal turnover in species composition within the wet season (March and December, R  =  0.889), and from spring (September) to summer (December) (R  =  1.000) and winter (June) to spring (September) (R = −0.556) (Fig. 2C). For millipedes (Fig. 2D), the only strong separation between seasons was between spring (September) and summer (December) (R  =  0.907). Ant species composition was strongly separated between spring (September) and summer (December) (R  =  0.796), but temporal turnover was also evident between autumn (March) and summer (December) (R  =  0.685) (Fig. 2E).

### Sampling Method Efficiency and Effectiveness

**Figure 3:**
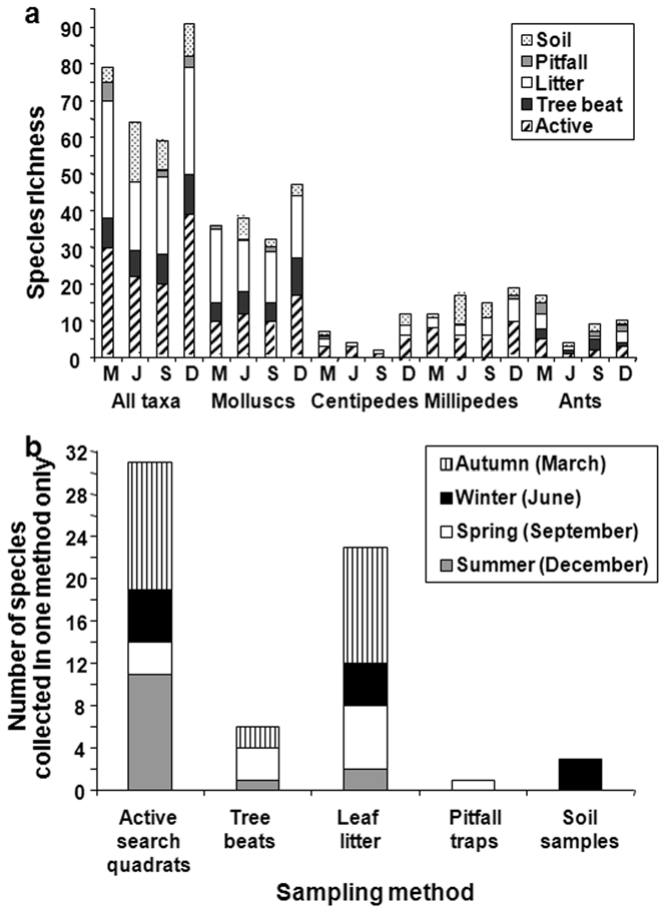
The effect of sampling method on species richness of different taxa in different seasons. (A) The contribution of different sampling methods to species richness counts (n  =  3 forests) for all taxa and individual taxa in different seasons (M  =  March (autumn), J  =  June (winter), S  =  September (spring) and D  =  December (summer)). (B) The number of species unique to one sampling method in each season (month).

The five different sampling methods used contributed unequally to species richness (Fig. 3B). Tree beats were important for collecting live snails and ants, but did not target any other taxa. Pitfall traps and soil samples performed poorly. Active search quadrats and leaf litter samples were the sampling methods that collected the greatest number of species collected by one method only in each month (Fig. 3A), and far outperformed the other three sampling methods in terms of number of species collected.

The mean efficiency (calculated as species per person hour) for soil samples was 1.0, pitfall traps was 0.2, litter samples was 2.6, active search quadrats was 2.6 and tree beats was 1.4 species per person hour respectively.

### Assessment of Taxa as Biodiversity Surrogates and Indicators

When assessing the potential of the different taxa as biodiversity surrogates and indicators of disturbance, molluscs scored highest followed by millipedes (Table 2). Centipedes scored lowest, with several of the categories indicating problems with the use of this taxon (Table 2). However, none of the taxa proved to be good surrogates for the underlying diversity (all taxa excluding the target taxon) in terms of species richness, with all relationships being non-significant (Linear Regression: molluscs: F_1,8_  =  0.983, P  =  0.351; earthworms: F_1,8_  =  0.008, P  =  0.933; centipedes: F_1,8_  =  0.521, P  =  0.616; millipedes: F_1,8_  =  0.778, P  =  0.404; and ants: F_1,8_  =  0.003, P  =  0.961).

**Table 2:** Potential of the different taxa as biodiversity surrogates and indicators of disturbance evaluated according to the criteria and scale provided by Summerville *et al.* [6], with endemism added.

Taxon	Diverse fauna (in forests)^a^	Well known taxonomy^b^	Easy to identify^c^	Well known natural history^d^	Readily surveyed^e^	High ecological fidelity (forests)^f^	Endemism^g^	Total score^h^
Ants	++	++	+	+	+++	++	+	12 (11)
Onychophorans	+	+	+	+++	+	++	++	11 (9)
Centipedes	++	+	+	+	++	+	+	9 (8)
Millipedes	+++	++	++	+	+++	+++	+++	17 (14)
Earthworms	++	+	+	+	+	+++	+++	12 (9)
Molluscs	+++	+++	+++	++	+++	+++	++	19 (17)

a In South Africa: <20  =  +; 21–50  =  ++; >50  =  +++.

b Well known taxonomy: % of species identifiable to species level by expert: >50%  =  +; 50–75%  =  ++; >75%  =  +++ (based on material collected in this study).

c Resources available for identification by non-expert: none  =  +; some but incomplete/difficult to use  =  ++; good  =  +++.

d Well known natural history: information on life history, diet, habitat available for taxon: none  =  +; some but incomplete  =  ++; good general knowledge  =  +++.

e Readily surveyed: require specialised sampling  =  +; require at least one specialised method  =  ++; easily sampled as part of general survey  =  +++.

f High ecological fidelity: species occur in both forest and matrix  =  +; most species restricted to forest  =  ++; all species limited to forest  =  +++.

g Endemism: <10% of species regional endemics (considering entire SA fauna)  =  +; 10–30% regional endemics  =  ++; >30% regional endemics  =  +++.

h Numbers in parentheses indicate Summerville *et al.* [6] score excluding endemism.

### Assessment of Molluscs as Indicators of Disturbance

Since molluscs best met Summerville *et al.* [6]'s criteria, we tested their ability to reflect disturbance (fire history), and to act as surrogates for the responses of other taxa to this disturbance, in forest patches at Royal Natal National Park.

**Figure 4:**
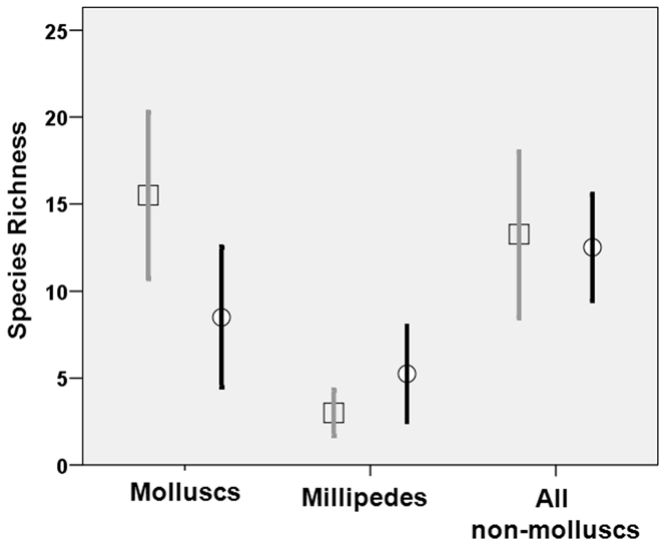
Effect of disturbance (fire treatment: unburned  =  grey line and squares; burned  =  black line and circles) on species richness of molluscs, millipedes, and all non-mollusc taxa combined (earthworms, centipedes, millipedes, and ants). Data are mean ±95% Confidence limits.

The species richness for molluscs was significantly lower in burned than unburned forest patches (ANOVA: F_1,6_  =  12.73; P  =  0.012) (Fig. 4). However, species richness of all non-molluscs was not significantly different between the burn treatments (F_1,6_  =  0.179, P  =  0.687). The species richness of millipedes was marginally non-significantly different across the treatments (F_1,6_  =  0.561, P  =  0.055). Therefore, in terms of species richness, molluscs were the most effective indicator of fire history.

Seven mollusc species had lower abundance in the burned forests, six species were found only in unburned forest, and two only in burned forests (Table 3). In contrast, no millipedes were found only in unburned forest, and three millipedes were found only in burned forest. A micromollusc, *Hydrocena noticola*, was the only species found in all four unburned forests, and in none of the burned forests. Of the larger species (shell length ±8 mm), *Macroptychia africana*, which was found in three of the four unburned forests and none of the burned forests, may be the most appropriate species for monitoring disturbance. Other potential candidates were *Nata vernicosa* and *Trachycystis subpinguis*.

**Table 3:** The effect of disturbance (fire history) on forest mollusc species at Royal Natal National Park.

		Abundance a	
Family	Species	Unburned	Burned
Hydrocenidae	*Hydrocena noticola* Benson, 1856	103	0
Pupillidae	*Lauria dadion* (Benson, 1864)	9	1
Orculidae	*Fauxulus glanvilleanus (darglensis)* (Ancey, 1888)	46	6
Orculidae	*Fauxulus mcbeanianus* Melville and Ponsonby, 1901	18	2
Vertiginidae	*Pupisoma harpula* (Reinhardt, 1886)	1	0
Vertiginidae	*Truncatellina sykesii* (Melville and Ponsonby, 1893)	11	0
Clausiliidae	***Macroptychia africana* (Melville and Ponsonby, 1899)** b	43	0
Streptaxidae	*Gulella juxtidens* (Melville and Ponsonby, 1899)	67	3
Streptaxidae	*Gulella mariae* (Melville and Ponsonby, 1892)	1	0
Streptaxidae	*Gulella* sp.	5	0
Rhytididae	***Nata vernicosa* (Krauss, 1848)**	8	1
Valloniidae	*Acanthinula* sp.	19	4
Charopidae	*Afrodonta novemlamellaris* (Burnup, 1912)	13	1
Charopidae	*Trachycystis contabulata* Connolly, 1932	2	9
Charopidae	*Trachycystis ectima* (Melville and Ponsonby, 1899)	46	61
Charopidae	*Trachycystis glanvilliana* (Ancey, 1893)	0	4
Charopidae	*Trachycystis rudicostata* Connolly, 1923	41	14
Charopidae	***Trachycystis subpinguis* Connolly, 1922**	23	4
Helicarionidae	*Kaliella euconuloides* Melville and Ponsonby, 1908	23	2
Euconulidae	*Afroconulus diaphanus* (Connolly, 1922)	0	5
Urocyclidae	***Sheldonia transvaalensis* (Craven, 1880)**	9	21

a Abundance scores are based on active search quadrat, litter sample and tree beat data.

b Species in bold are not micromolluscs [48].

**Figure 5:**
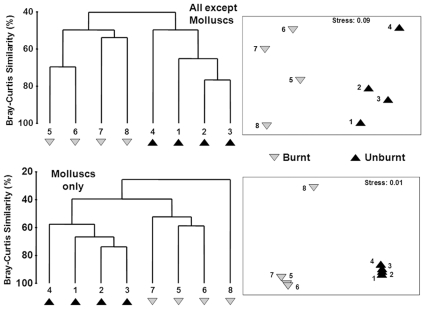
Effectiveness of the mollusc community as an indicator of the effect of disturbance (fire history) on the balance of taxa (earthworms, centipedes, millipedes, and ants).

The mollusc community at the unburned sites clustered out distinctly from those at the burned sites (ANOSIM: R  =  0.719) (Fig. 5B). While the non-mollusc community (earthworms, centipedes, millipedes and ants) also clustered out distinctly in terms of sites with different fire histories (R  =  0.875) (Fig. 5A), the clustering was a lot less distinct than that for molluscs (excluding site 8, which although clustering separately from the unburned sites was not closely clustered with the burned sites) (Fig. 5A). In addition, there was a lack of concordance in the communities of molluscs at burned sites relative to those at unburned sites (PROTEST: m*^2^*  =  0.431, P  =  0.685). Therefore, mollusc community structure itself may serve as a strong indicator of disturbance, in this case fire history.

## Discussion

We provide here an additional example of the stepwise practice for selecting surrogates and indicators [6], [15]. An important component of this process is independent testing of the indicator for monitoring, and because our test of this involved a discrete and easily identifiable disturbance event, the results should be relatively robust. Such testing follows the recommendations of Pocock and Jennings [14], who highlighted that variable sensitivity of taxa to different kinds of disturbance makes them more or less effective as indicators. Note that, as in our study, even within the same higher-level taxon, species may respond differently to a particular disturbance, and each species needs to be tested [14].

An important additional component that we have added to the selection process of Rohr *et al.* [15], is evaluating temporal (seasonal) effects. Some taxa will be more or less abundant through the year, as they are more sensitive to temperature or moisture changes, or have different life stages that are only present in certain seasons. We highlight that, as part of the initial survey step, sampling should be conducted at different times of the year to identify (1) the best time of the year to sample, and (2) the taxa least likely to vary with season. This is important both in carrying out biodiversity assessments or inventories, and for monitoring.

Mollusc species richness was remarkably similar across seasons, although, as with other taxa, there were significant changes in community structure. The stability of the mollusc richness may be explained by the persistence and presence of dead shells, even if the live animals are not active. Any use of community assemblages should therefore control for season, and, in general, March (autumn/late summer, wet season) was the best time for sampling across taxa. Note that we only present data for a single year, and diversity may be more variable over multiple years (*e.g.* carabid beetles [30]). We recommend longer term survey work to assess temporal stability from year to year.

None of our taxonomic groups were good predictors of the balance of diversity, and a similar lack of congruency has been found by others [16], [31]–[39], but see, *e.g.* [40]. This is despite the fact that our study focussed in one vegetation type Afrotemperate forest [27], and in a limited geographic region, where we would not expect biogeographic factors to confound relationships [see 39]. Note that local biogeographic processes such as forest area and within-valley isolation were not important drivers of our community assemblages [41].

We highlight in this work the use of community structure of a particular taxon being used as a surrogate of diversity changes in response to disturbance, rather than simply species richness [8]. Given advances in statistical analyses using ordination techniques [15], robust interpretations of assemblage changes could prove valuable. This is particularly so for the hyperdiverse invertebrates, where a single species, or species richness *per se*, may not be appropriate, especially for surrogates in conservation planning.

While several studies have investigated invertebrate sampling methods [*e.g.* 15], [42]–[44], there are still no generally accepted, standardized sampling methods or protocols for different invertebrate taxa. The effectiveness of different sampling methods may also vary temporally depending on the activity patterns of the target taxa [45]. By focussing our monitoring on a particular indicator taxon, we can also focus our sampling effort. We consider three major aspects: (1) effectiveness at sampling the target taxon; (2) ease of implementation by managers in relatively remote areas; and (3) impact on the [protected] fauna of a reserve that is repeatedly sampled for monitoring. Considering these three aspects, we recommend for molluscs a combination of quantified litter sampling and timed active searching in restricted quadrats. This combination was effective, can be implemented in one short visit, and allows the release alive of repeatedly sampled species. Passive techniques, such as pitfall traps, should be critically assessed [see also 2] when considering monitoring programmes as they: (1) are not as effective as active searching techniques [*e.g.* 43]; (2) require managers to transport more equipment to remote sites, and require repeat visits to collect samples; and (3) kill (potentially large numbers of) both the target taxon and a large bycatch unnecessarily [see also 46], which can compound with repeated sampling; a problem when sampling in protected areas conserving threatened species, especially in small, patchy habitats such as the Drakensberg Afrotemperate forests. When used for monitoring purposes, it may be necessary to differentiate freshly dead from very old shells, to ensure that the current community is being sampled (we do not know the decomposition rates of shells in our habitat, or the effect of fire on old shells).

Besides the other attributes favouring molluscs as surrogates or indicators, they have high inherent conservation value, with high local species richness, and high levels of endemism, with relatively narrow distribution ranges [*e.g.* 47 and references therein], [48]. This would make snails particularly advantageous as fine filter features in conservation planning. Millipedes, the second most appropriate taxon, similarly have high inherent conservation value [4].

Ants have typically been used as indicators for a wide range of aspects [reviewed in 49]. In our analysis they were not as effective as surrogates or indicators relative to snails or millipedes. One reason for this may be the relatively low number of species sampled. However, they may not be suitable within this particular habitat, or within the spatial scale of the study.

Our study habitat type, *i.e.* Afrotemperate forest, was constant, but our results indicated wide variation in species richness across forest patches, as well as shifts in community structure (for another such example with snails see [48]). This means that setting targets and selecting habitats for management and conservation based purely on vegetation would be likely to miss potentially important species or communities. Under these circumstances, using identified species of the taxa included in this study will improve the fine-scale selection and prioritization of forest patches. We recommend that both this aspect of invertebrate conservation, and the use of molluscs as indicators of disturbance, be evaluated more broadly.

Our results also emphasise the effect of fire within these Afrotemperate patches on invertebrate communities, and careful attention needs to be paid to management of fire within these systems [see also 29].

## Methods

The Maloti-Drakensberg Bioregion experiences summer rainfall, with 70% of the annual precipitation in the austral summer (November to March) [50]. Median rainfall values ranging across the Bioregion in the months that seasonal sampling took place are as follows: March, 100 −140 mm; June, <5 mm; September, 20–60 mm; and December, 120–160 mm. Mean annual temperature in the Drakensberg is 16°C, with mean daily maximum temperatures ranging from 26.7°C in summer to 15.6°C in winter [50]. Temperatures can drop to below zero in winter.

We sampled three Afrotemperate forest patches (sites) at Injisuthi (Appendix S1) in March (late summer, wet season), June (winter, dry), September (spring, dry) and December (mid-summer, wet) 2004, to assess the effect of seasonal changes on invertebrate species richness and community structure. To increase our power for assessing surrogacy of molluscs or millipedes for overall diversity, we sampled an additional two forests at Injisuthi (total five), four (unburned) forests at Royal Natal and one forest at Cathedral Peak between November 2004 and January 2005 (Appendix S1).

We experimentally tested our conclusions for monitoring based on the Injisuthi data by contrasting four burned forests (in Devil's Hoek valley) with four unburned forests (in Thukela Gorge valley) at Royal Natal National Park (Appendix S1). According to Ezemvelo KZN Wildlife fire records, Devil's Hoek valley last burned in January 2003, 22 months prior to sampling, while Thukela Gorge forests had not burned during the same invasive fire and neither valley was burned again until after sampling took place at Royal Natal in November 2004.

The same sampling methods, sampling intensity and taxa were used in each forest patch at each sampling event. We collected six 0.3 L soil cores, 5 m apart in a straight line. Soil samples were kept in a cool place (refrigerator when possible) and processed within 14 days of collection. We placed soil samples in Berlese funnels for 48 h to extract invertebrates, after which we checked the soil in the funnel for large invertebrates unable to crawl through the 1 mm^2^ gauze.

We set six pitfall traps (plastic 0.125 L screw top jars, 75 mm deep and 40 mm diameter) per forest into the holes from the soil samples. We filled traps with a glycerol-ethanol mixture and collected these pitfall traps after six days.

To specifically target micro-molluscs (adults have shells <5 mm diameter – [51]), we collected two 2 L leaf litter samples from each forest from areas that had not been disturbed by the team. The litter sample was collected from a single point covering about 0.25 m^2^, taken at a set distance along a randomly placed transect to avoid collector bias. We collected and identified both live and dead molluscs, as well as all other target taxa. Litter samples were sorted by hand within 48 h of collection. Snails were drowned in water then preserved in 70% ethanol.

One set of five contiguous 2×2 m quadrats, covering an area of 20 m^2^ on undisturbed ground was sampled in each forest. We searched all leaf litter, rocks, logs, vegetation below 0.5 m and the top 50 mm of soil, covering the entire area thoroughly for target taxa. It took one person approximately 210 min to search 20 m^2^.

Although sampling was focussed on ground-dwelling taxa, some molluscs and ants also occur in trees. We beat ten under-storey trees per forest, selected based on their accessibility. We struck one branch of each tree five times with a large wooden stick, and collected all molluscs and ants that fell onto a white, flat, round, cotton, 0.7 m diameter collecting net.

For each 2×2 m quadrat and tree beat sampling, we collected representative samples in the field, recorded the number of individuals of target taxa, and released live extra specimens where we sampled a large number of individuals, and where species were readily recognisable. Earthworms were prepared as follows: each individual was rinsed in water, preserved in a weak (40%) solution of ethanol, allowed to dry for four minutes and then fixed in 4% formalin. All other invertebrates were frozen and then preserved in 70% ethanol.

Target taxa were sorted to morphospecies in the laboratory and identified to species by respective taxonomic experts as follows: molluscs, Dr Dai Herbert (Natal Museum); earthworms, Dr Danuta Plisko (Natal Museum); onychophorans, centipedes and millipedes, Prof. Michelle Hamer (UKZN/SANBI); and ants (wingless workers only, identified to morphospecies only), Dr Hylton Adie (UKZN). The reference collection is lodged in the Natal Museum, Pietermaritzburg for use in future studies.

Sampling intensity was relatively low because of the small size of the forests and the need to minimize disturbance in the forests. Sample-based species-accumulation curves [52] were plotted for each site using PRIMER [53]. We calculated effort as the approximate number of person hours taken for field sampling and processing of each replicate of each sampling method. We plotted species presence/absence against this effort using 999 permutations. Species-accumulation curves for all methods combined approached an asymptote across sites within a sampling month (Appendix S2). Observed species richness of tropical arthropods rarely reaches an asymptote, even with intensive sampling [52]. To avoid pseudoreplication [54], data from replicates taken at each site were combined into a single datum per sample method per site. These data indicate that we sampled a substantial portion of the diversity of our target taxa using our methods and effort.

### Determination of Suitable Sampling Methods for Use in Biodiversity Assessment and Monitoring

To compare the contribution of different sampling methods to species richness counts in different seasons, we plotted species richness from each sampling method in each month for all taxa combined and separately for each target taxon. To determine which sampling method(s) were most suitable for targeting rare species and species with relatively short adult stages or short periods of surface activity, we noted the number of species collected by only one sampling method in each month for all taxa combined. We calculated efficiency of each sampling method (*i.e.* sampling effort) as the total number of species recorded in three forests combined, divided by the number of person hours required for sampling and processing. We calculated efficiency (species per person hour) for each sampling method in each month, and mean efficiency for each sampling method as the mean of four months.

### Evaluation of Suitability of Taxa for Use as Biodiversity Surrogates and Indicators of Disturbance

Taxa were assessed according to the criteria and scale presented by Summerville *et al.* [6]. Data on the different taxa were obtained from experts, from the literature [17]–[21], [55]–[57], or from relevant websites [58].

We assessed potential for surrogacy for overall diversity by regressing the diversity excluding a taxon against the diversity for that taxon [6]. For this analysis we used data collected in the same way in ten forests across four study sites: Royal Natal (4); Cathedral Peak (1) and Injisuthi (5). Residuals were normally distributed (Kolmogorov-Smirnov test: all P>0.05).

### Monitoring Using Our Recommendations: Assessment of Response to Environmental Disturbance

Given that we had identified molluscs as the taxon with the highest indicator potential, we focused this analysis on molluscs. We compared total and mean mollusc species richness of forests between unburned and burned valleys at Royal Natal National Park using analysis of variance (ANOVA). We compared mollusc species richness measured by quadrat, litter sample or tree beating to determine methods required to sample mollusc species richness for monitoring purposes. To determine whether mollusc species richness reflected the influence of fire on other taxa, we compared mollusc data with centipede, millipede and ant data.

We assessed the effectiveness of the mollusc community in reflecting differences associated with disturbance (fire history) by performing a Bray-Curtis similarity matrix using square-root transformed abundance data in PRIMER. We then performed an MDS plot and cluster analysis to assess differences among sites with different histories. If molluscs are a good indicator of disturbance, their communities should cluster according to fire history. In addition, we assessed whether the mollusc community reflected similar changes in the communities of other taxa by performing the same analysis for all taxa excluding molluscs. As an additional analysis, we performed a Procrustean randomisation test using PROTEST [59] which contrasts two community matrices in a more robust manner than Mantel tests [59]. Here we contrasted the community of molluscs at the four burned forests relative to the community of molluscs at the four unburned forests. In addition, we contrasted the community of all non-mollusc taxa in the burned relative to the unburned forests to assess if these were similarly (as with molluscs) different from each other.

## Supporting Information

Appendix S1 Forest details with an indication of which forests were used for specific analyses. MAP  =  mean annual precipitation.
(0.05 MB DOC)

Appendix S2 Sampling saturation for the Injisuthi seasonal sampling. We present randomized species-accumulation curves of all target taxa combined in each month that seasonal sampling took place: (A) autumn (March), (B) winter (June), (C) spring (September), and (D) summer (December). The x-axes represent the number of person hours taken to collect and process each sampling replicate.
(0.06 MB DOC)
