# Supplementary material for: Step Process for Selecting and Testing Surrogates and Indicators of Afrotemperate Forest Invertebrate Diversity
Source: PLoS One. 2010 Feb 9;5(2):e9100. doi: 10.1371/journal.pone.0009100 (PMC2817749; doi:10.1371/journal.pone.0009100)
Supplement: Appendix S1 — Forest details with an indication of which forests were used for specific analyses. MAP = mean annual precipitation. (0.05 MB DOC) [file pone.0009100.s001.doc]

| **Reserve** | **Site** | **Latitude** | **Longitude** | **Area** | **Altitude** | **MAP** | **Analysis** |
| --- | --- | --- | --- | --- | --- | --- | --- |
|  |  |  |  | **(ha)** | **(m asl)** | **(mm)** |  |
| Injisuthi | 1 | 29^o^ 7' 10.9200" S | 29^o^ 26' 9.2400" E | 1.41 | 1500 | 1022 | Season; Surrogacy |
| Injisuthi | 2 | 29^o^ 6' 28.4400" S | 29^o^ 26' 0.2394" E | 2.46 | 1650 | 1076 | Season; Surrogacy |
| Injisuthi | 3 | 29^o^ 6' 35.9994" S | 29^o^ 26' 20.0400" E | 5.28 | 1600 | 1022 | Season; Surrogacy |
| Injisuthi | 4 | 29^o^ 6' 35.9994" S | 29^o^ 26' 20.4000" E | 3.92 | 1600 | 1022 | Surrogacy |
| Injisuthi | 5 | 29^o^ 6' 27.7200" S | 29^o^ 25' 46.9200" E | 2.41 | 1650 | 1076 | Surrogacy |
| Royal Natal | 1 | 28^o^ 43' 53.7600" S | 29^o^ 54' 48.2394" E | 12.50 | 1700 | 1327 | Surrogacy; Disturbance |
| Royal Natal | 2 | 28^o^ 43' 49.7994" S | 29^o^ 55' 4.7994" E | 14.00 | 1600 | 1327 | Surrogacy; Disturbance |
| Royal Natal | 3 | 28^o^ 43' 45.1194" S | 29^o^ 55' 16.3194" E | 7.64 | 1620 | 1327 | Surrogacy; Disturbance |
| Royal Natal | 4 | 28^o^ 43' 39.3594" S | 29^o^ 55' 25.3200" E | 4.26 | 1600 | 1327 | Surrogacy; Disturbance |
| Royal Natal | 5 | 28^o^ 42' 36.7200" S | 29^o^ 55' 25.5194" E | 35.70 | 1700 | 1225 | Disturbance |
| Royal Natal | 6 | 28^o^ 42' 37.7994" S | 29^o^ 55' 27.8394" E | 12.00 | 1620 | 1225 | Disturbance |
| Royal Natal | 7 | 28^o^ 42' 32.0394" S | 29^o^ 55' 43.3194" E | 11.40 | 1620 | 1142 | Disturbance |
| Royal Natal | 8 | 28^o^ 42' 39.9594" S | 29^o^ 55' 52.6800" E | 8.28 | 1550 | 1142 | Disturbance |
| Cathedral Peak | 3 | 28^o^ 57' 29.1600" S | 29^o^ 13' 14.5200" E | 21.8 | 1550 | 1167 | Surrogacy |
