# Supplementary figures and images for: Step Process for Selecting and Testing Surrogates and Indicators of Afrotemperate Forest Invertebrate Diversity
Source: PLoS One. 2010 Feb 9;5(2):e9100. doi: 10.1371/journal.pone.0009100 (PMC2817749; doi:10.1371/journal.pone.0009100)

**
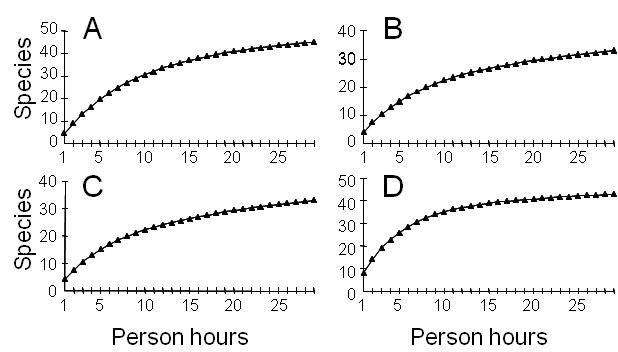
**

Supplement: Appendix S2 — Sampling saturation for the Injisuthi seasonal sampling. We present randomized species-accumulation curves of all target taxa combined in each month that seasonal sampling took place: (A) autumn (March), (B) winter (June), (C) spring (September), and (D) summer (December). The x-axes represent the number of person hours taken to collect and process each sampling replicate. (0.06 MB DOC) [file pone.0009100.s002.doc]
